# A Class of Protein-Coding RNAs Binds to Polycomb Repressive Complex 2 and Alters Histone Methylation

**DOI:** 10.3389/fonc.2021.739830

**Published:** 2021-11-05

**Authors:** Meijian Liao, Xiaolin Sun, Shoucui Gao, Yaou Zhang

**Affiliations:** ^1^ Department of Pathology, Xuzhou Medical University, Xuzhou, China; ^2^ State Key Laboratory of Chemical Oncogenomics, Graduate School at Shenzhen, Tsinghua University, Shenzhen, China; ^3^ School of Life Sciences, Tsinghua University, Beijing, China; ^4^ Key Lab in Healthy Science and Technology, Division of Life Science, Graduate School at Shenzhen, Tsinghua University, Shenzhen, China; ^5^ Open FIESTA Center, Graduate School at Shenzhen, Tsinghua University, Shenzhen, China

**Keywords:** TMEM117, PRC2 complex, protein-coding RNA, H3K27me3 modification, LncRNA

## Abstract

Polycomb repressive complex 2 (PRC2) is a multi-subunit protein complex mediating the methylation of lysine 27 on histone H3 and playing an important role in transcriptional repression during tumorigenesis and development. Previous studies revealed that both protein-coding and non-coding RNAs could bind to PRC2 complex. However, the functions of protein-coding RNAs that bind to PRC2 complex in tumor are still unknown. Through data mining and RNA immunoprecipitation (RIP) assay, our study found that there were a class of protein-coding RNAs bound to PRC2 complex and H3 with tri-methylation on lysine 27. The Bayesian gene regulatory network analysis pointed out that these RNAs regulated the expression of PRC2-regulated genes in cancer. In addition, gene set enrichment analysis (GSEA), gene ontology (GO) analysis, and weighted gene co-expression network analysis (WGCNA) also confirmed that these RNAs were associated with histone modification in cancer. We also confirmed that MYO1C, a PRC2-bound transcript, inhibited the modification level of H3K27me3. Further detailed study showed that TMEM117 regulated TSLP expression through EZH2-mediated H3K27me3 modification. Interestingly, the RNA recognition motif of PRC2 complex might help these RNAs bind to the PRC2 complex more easily. The same regulatory pattern was found in mice as well.

## Introduction

Tri-methylation of the H3 histone at Lys 27 (H3K27me3) is critical for long-term transcriptional repression, playing an important role during tumorigenesis and development (1). The modification of H3K27me3 catalyzed by the polycomb repressive complex 2 (PRC2) is well studied. PRC2 complex contains three major proteins: EZH2, SUZ12, and EED. The EZH2 catalytic subunit, a methyltransferase, catalyzes tri-methylation of histone H3 at Lys 27 leading to the epigenetic repression of its target gene (2). SUZ12 protein acts as stability factor for PRC2 complex and influences the catalytic activity of EZH2 (3). EED protein can bind to histone with an H3K27me3 modification through its WD40 domain, enhancing the catalytic activity of EZH2 protein (4). All three of them are closely related to the functions of cancer cell proliferation, apoptosis, and metastasis (5–8).

Recently, the mechanism of PRC2 complex recruitment to chromosomes has been widely studied. However, how PRC2 complex is recruited to the specific loci of genome remains unclear. Previous studies have proposed several potential mechanisms. Some investigations found that PRC2 complex has a preference to occupy CpG-rich regions (9). In addition, its associated factor, JARID2, is a DNA-binding factor that has been proposed to either enhance or repress the binding of PRC2 complex to its target genes (10, 11). Besides, evidences indicate that some long non-coding RNAs (lncRNAs) act as potential guides, tethering PRC2 complex to specific genomic loci. For example, Hox transcript antisense RNA (HOTAIR) interacts with PRC2 complex and guides histone H3K27me3 modification (12). Additionally, metastasis-associated lung adenocarcinoma transcript 1 (MALAT-1), KCNQ1 opposite strand/antisense transcript 1 (kcnq1ot1), and other lncRNAs have been found to interact with EZH2 protein and regulate histone H3K27me3 modification (13, 14). However, it is controversial whether the interaction between lncRNAs and PRC2 complex is specific. Some investigations point out PRC2 complex has high affinity for short tracts of G’s and G-quadruplexes (15, 16). In addition, the two-hairpin secondary structure is considered to be the RNA recognition motif of PRC2 complex (17). Nevertheless, several unbiased genome-wide studies propose that RNAs bind to PRC2 complex promiscuously, indicating that PRC2 complex interacts with RNAs in general and does not have a recognition motif for a specific RNA sequence (18). Recently, it has been found that besides lncRNAs, protein-coding RNAs are capable of binding to PRC2 complex, suggesting that protein-coding RNAs may play a role in PRC2 complex recruitment and chromatin modification (19, 20). Nevertheless, there is no report on the functions of the protein-coding RNAs binding to PRC2 complex.

Therefore, we ask whether protein-coding RNAs regulate histone methylation through binding to PRC2 complex, just like lncRNAs. In this manuscript, we show that there is a class of protein-coding RNAs that binds to PRC2 complex and epigenetically regulates gene expression. The RNA recognition motif of PRC2 complex may help this class of RNAs more easily bind to PRC2 complex and regulate histone methylation. Apart from being a messenger between DNA and protein, we first propose and demonstrate that protein-coding RNAs are able to function similarly to lncRNAs, which bind to PRC2 complex and regulate histone H3K27me3 modification.

## Materials and Methods

### Cell Culture and Transfection

HeLa (ATCC, CCL-2) and MEF (ATCC, SCRC-1045) cells were purchased from ATCC. Cells were cultured with modified Eagle’s medium (Thermo Fisher Scientific, 12100046) containing 10% fetal bovine serum (FBS) (Biowest, S1810) in 5% CO_2_ incubator. SiRNAs were transfected using Lipofectamine 3000 as described in the manufacturer’s protocol. The sequences of siRNAs are shown in **Table S1**.

### Datasets

The University of California Santa Cruz UCSC Xena database (https://xena.ucsc.edu) is an online tool for visualization of gene expression profiles and clinical data (21). The gene expression profiles of cervical cancer (CESC), acute myeloid leukemia (LAML), bladder cancer (BLCA), breast cancer (BRCA), head and neck cancer (HNSC), liver cancer (LIHC), lung adenocarcinoma (LUAD), pancreatic cancer (PAAD), prostate cancer (PRAD), and stomach cancer (STAD) were collected in the data hubs of UCSC Xena. The set of Illumina HiSeq (version 2017-10-13) in each type of tumor was downloaded. The gene expression profiles of mice, chromatin immunoprecipitation (ChIP)-seq of mice, RNA immunoprecipitation (RIP)–ChIP, fRIP-seq, and par-CLIP-seq data were downloaded from the Gene Expression Omnibus (GEO) datasets (https://www.ncbi.nlm.nih.gov/geo/). The ChIP-seq data of seven human cell lines were downloaded from the Encyclopedia of DNA Elements (ENCODE) database (https://www.encodeproject.org/). The detailed information of every datasets is shown in **Table S2**.

### RNA Immunoprecipitation Assay

First, cells from 10-cm culture dish were washed with 5 ml of cold phosphate-buffered saline (PBS) twice, and then 1 ml of polysome lysis buffer (10 mM of KCl, 5 mM of MgCl_2_, 10 mM of HEPES, pH 7.0, 0.5% NP-40, 1 mM of DTT, 100 U/ml of RRI, 20 μl/ml of PIC, and 2 mM of vanadyl ribonucleotide complex solution) was added. Subsequently, cells were scraped and transferred to a fresh microtube on ice and centrifuged at 14,000 ×*g* for 15 min at 4°C. The supernatant was collected; 2 μg of primary antibody (EZH2, 21800-1-AP; H3K27me3, ab192985) was added and incubated on an end-to-end rotator overnight at 4°C; 2 μg of IgG (abcam, ab171870) was used as negative control. Dynabeads protein G measuring 40 μl (Thermo Fisher Scientific, 88848) was added and incubated for another 4 h. The complex was washed four times with 500 μl of polysome lysis buffer. Subsequently, the complex was re-suspended using 100 μl of polysome lysis buffer containing 0.1% sodium dodecyl sulfate (SDS) and 30 μg of Proteinase K and incubated at 50°C for 30 min. The supernatant was collected, 100 μl of phenol–chloroform–isoamyl alcohol was added, and then the water phase was collected. Subsequently, 5 μl of linear polyacrylamide, 10 μl of 3 M sodium acetate, and 250 μl of ethanol were added and then precipitated at −20°C overnight. Finally, the RNA was collected and analyzed using RT-PCR.

### Bayesian Gene Regulatory Network Analysis

First, we identified PRC2-bound transcripts through analyzing the RIP–ChIP or par-CLIP-seq data of EZH2 and SUZ12 proteins (GSE16226 and GSE49435). The genes with H3K27me3, EZH2, and SUZ12 occupation on their promoter region (−5 kb, +1 kb) were considered to be regulated by PRC2 complex. Therefore, we secondly analyzed the PRC2-regulated genes through analyzing the ChIP-seq data of H3K27me3, SUZ12, and EZH2 in seven human cell lines of ENCODE database or in mouse cells of GEO. Finally, the Bayesian network was performed on the expression profiles of PRC2-bound transcripts and PRC2-regulated genes mentioned above. Briefly, the RIP–ChIP and ChIP-seq data were download and cleaned using trimmomatic [trimmomatic PE -threads -phred33 -basein <input> -baseout <paired output 1> <unpaired output 1> <paired output 2> <unpaired output 2>] (22). Then the RIP-seq data were aligned using hisat2 [hisat2 –phred33 -x <hisat2-idx> -1 <m1> -2 <m2> -S <output. sam>] and mapped using StringTie [stringtie <aligned_reads.bam> -p 25 –G <indexes> -B –o <output. gtf>] (23, 24). Subsequently, the ChIP-seq data were aligned using bowtie2 [bowtie2-build <reference_in> <bt2_base>] and mapped using macs2 [macs2 callpeak -t <text.bam> -c <input.bam> -g hs -B -f BAM -n test -q 0.01] (25, 26). Then mRNA expression profiles of CESC, LAML, BLCA, BRCA, HNSC, LIHC, LUAD, PAAD, PRAD, and STAD tissues in The Cancer Genome Atlas (TCGA) database were normalized together using z-score. Furthermore, the Bayesian gene regulatory network analysis was performed using pcalg package (version 2.6-7) of R software (27). The regulatory between control genes and PRC2-regulated genes was also studied. Two unbiased ways were used to select control genes. The ways to select control genes based on their expression are as follows: 1) all genes were ranked according to their average mRNA levels in all samples of the above 10 cancers. 2) Forty-five genes with their mRNA expression nearest to the 45 PRC2-bound transcripts were defined as control genes. Another method was selecting genes randomly: 1) all genes were ranked according to their name in the Excel; 2) using the rand function to obtain a set of random numbers and corresponding them to the gene name; 3) all genes were ranked according to the random numbers, and the top 45 genes were considered as control genes. Please see the **Supplementary Methods** for the R scripts of Bayesian gene regulatory network.

### Library of Integrated Network-Based Cellular Signatures Analysis

Library of Integrated Network-Based Cellular Signatures (LINCS) project studies how cells respond to various genetic and environmental stressors and helps us understand the cell pathways (28, 29). An online tool “query” of LINCS (https://clue.io/query) was performed to evaluate the gene signature patterns. The 48 PRC2-regulated genes were defined as PRC2-regulated signature and put into the upregulated gene sets of “query” tool. The similarity score would be calculated by “query” tool online. Finally, the genes were ranked according to the similarity score. The control genes were selected based on their mRNA expression nearest to PRC2-bound RNAs, as described in the Bayesian gene regulatory network analysis.

### Gene Set Enrichment Analysis

The gene set enrichment analysis (GSEA) was performed using GSEA v4.1.0 software (30). The patients were divided into two groups according the median of mRNA expression of each gene. Gene sets were analyzed using “c5.all.v7.4.symbols.gmt” downloaded from MSigDB (http://www.gsea-msigdb.org/gsea/msigdb/index.jsp). One thousand permutations of each gene set were used. The pathways with *p* < 0.05 and false discovery rate (FDR) <0.25 were considered markedly enriched. All the histone modification-related pathways were displayed using heatmap.

### Gene Ontology Analysis

The gene ontology (GO) analysis was performed using the clusterProfiler package (version 3.18.1) of the R software (version 3.6.3) (31). The pvalueCutoff was set as 0.05, and the items with *p* < 0.05 were considered to have significant enrichment. The sub-ontologies of biological processes (BP) and molecular function (MF) were studied.

### Real-Time Quantitative PCR

Total RNA was isolated using RNA iso Plus (Takara, D9108B). Subsequently, reverse transcription was performed using ReverTra Ace qPCR RT Kits (TOYOBO, FSQ-101), and qRT-PCR was carried out using a SYBR^®^ Green Real time PCR Master Mix (TOYOBO, QPK-201). All samples were repeated in triplicate. Data were normalized to ACTB, and 2^−ΔΔCt^ was used to calculate the relative fold change. The primers of this study are shown in **Table S3**.

### Western Blotting Assay

Cultured cells in the six-well were extracted on ice using 200 μl of cell extraction buffer (50 mM of Tris-HCl, pH 8.0, 4 M of urea, and 1% Triton X-100) containing protease inhibitor mixture (Roche Diagnostics, 04693132001). Cell lysates were analyzed by Western blotting using EZH2 (CST, 5246, 1:1000), TMEM117 (ProteinTech, 21314-1-AP, 1:500), H3K27me3 (H3K27me3, ab192985, 1:1,000), and GAPDH (ProteinTech, 60004-1-Ig, 1:5,000) antibodies.

### Weighted Gene Co-Expression Network Analysis

The weighted gene co-expression network analysis (WGCNA) of the gene expression profiles of CESC was performed using the WGCNA package (version 1.67) of the R software (version 3.6.3) (32). The appropriate power value in the co-expression module construction was 0.8. Functional enrichment analysis of every co-expression module was performed by GO analysis using clusterProfiler package (version 3.15.1) (31). Only 40 PRC2-bound transcripts with their expression information were shown in CESC. The module–gene relationships were estimated using the correlation between modules and the expression of 40 PRC2-bound transcripts, or control genes. The control genes were selected randomly or based on their expression, as described in the Bayesian gene regulatory network analysis. The gene with absolute value of coefficient greater than 0.2 and *p* < 0.05 was considered to be related to the module. Finally, the proportion of related genes in each group of control genes was analyzed and compared with that in the PRC2-bound transcripts.

### Extraction of RNA in Nucleus and Cytoplasm

The nuclear and cytoplasmic protein extraction kit (Beyotime Biotechnology, P0027) was used to separate the RNA in nucleus and cytoplasm fraction. Briefly, cells from 10-cm culture dish were washed using 5 ml of PBS and then harvested. Subsequently, 800 μl of buffer A containing phenylmethylsulfonyl fluoride (PMSF) was added and vortexed vigorously for 5 s, and then the cells were incubated on ice for 15 min. Next, 40 μl of buffer B was added and vortexed vigorously for 5 s, and then the cells were incubated on ice for 1 min. Cells were centrifuged at 14,000×*g* for 5 min at 4°C, and then the supernatant was collected. The precipitation was washed three times using 500 μl of PBS each. The RNA in both supernatant and precipitation fraction was isolated using RNA iso Plus and then quantified using qRT-PCR assay.

### Fluorescence *in Situ* Hybridization and Immunofluorescence

The fluorescence *in situ* hybridization (FISH) assay was performed using TMEM117-specific probes (Biosearch Technologies). First, cells in six wells were fixed with 1 ml of 3% paraformaldehyde for 10 min at room temperature and then permeated for 7 min on ice using 1 ml of PBS containing 0.5% v/v Triton X-100. Cells were washed twice using 500 μl of 70% ethanol for 5 min each and then successively dehydrated in 500 μl of 80%, 95%, and 100% ethanol for 3 min each. Subsequently, 1 μl of RNA probe was added to incubate the cells overnight at 37°C. Then the cells were washed three times and each time for 5 min at 42°C using 500 μl 2× SSC. Subsequently, the cells were blocked with 1 ml of 3% bovine serum albumin (BSA) for 15 min and then incubated with EZH2 antibody (CST, 5246, 1:250) for 1 h at room temperature. The samples were washed three times using 1 ml of PBS and incubated with secondary rabbit antibody (CST, 4412, 1:250) for 1 h. Finally, the samples were stained with DAPI and imaged with an Olympus FV1000 confocal microscope.

### Chromatin Immunoprecipitation Assay

First, HeLa cells from 10-cm culture dish were washed with 1 ml of cold PBS and then fixed using 5 ml of 4% formaldehyde for 10 min on ice. Cells were harvested on ice and re-suspended with 600 μl of ChIP lysis buffer (50 mM of Tris-HCl, 5 mM of EDTA, 0.1% deoxycholated, 1% Triton X-100, 150 mM of NaCl, and proteinase inhibitor). Subsequently, cells were sonicated (10/20 s of pulse/pause, 18 cycles, and 30% power) and then centrifuged at 14,000 ×*g* for 15 min at 4°C, and the supernatant was collected. For the ChIP assay, cell lysates, 1 μg of ChIP grade H3K27me3 primary antibodies (abcam, ab192985), and 25 μl of Dynabeads protein G (Thermo Fisher Scientific, 88848) were mixed and incubated for 2 h at 4°C; 1 μg of IgG antibody (abcam, ab171870) was used as negative control. The complex was washed three times with 500 μl of PBS. To release immune complex, 40 μl of 10% Chelex-100 was added, and the suspension was collected. Finally, boiling the samples for 10 min and the DNA fractions were analyzed using qRT-PCR.

### Polycomb Repressive Complex 2 Recognition Motif Analysis

The MEME suite (https://meme-suite.org/meme/) is an online motif-based sequence analysis tool, helping biologists discover novel motifs. The fRIP-seq of EZH2 (GSE67963) and par-CLIP-seq of EZH2 (GSE49435) were used to study PRC2 recognition motif of humans and mice, respectively. The association between putative motifs and EZH2 or SUZ12-bound transcripts was analyzed using Poisson distribution (MATLAB), as described by Wang et al. (15).

### RNA-Pulldown Assay

HeLa cells from 10-cm culture dish were washed with 1 ml of cold PBS and then harvested using 0.5 ml of cell lysis buffer (10 mM of HEPES, pH 7.0, 200 mM of NaCl, 1% Triton X-100, 10 mM of MgCl_2_, 1 mM of DTT, and protease inhibitor). Subsequently, cells were sonicated (20/30 s of pulse/pause, 4 cycles, and 30% power). Cells were centrifuged at 14,000 ×*g* for 15 min at 4°C, and then the supernatant was collected. Yeast RNA (~0.5 μg/mg protein) was added and incubated on a rotation shaker for 20 min at 4°C. Cell lysates were centrifuged at 14,000 ×*g* for 15 min at 4°C, and then the supernatant was collected. The plasmids of TMEM117 with or without the RNA recognition motif mutation were construction using a pcDNA3.1 plasmid. Subsequently, the plasmids were linearized and transcribed *in vitro* for 1 h using a transcription kit (Thermo Fisher Scientific, AM1312). After transcription, the samples were boiled for 10 min, and poly-A tail was added to the 3′ end of RNA *in vitro* for 1 h using a tailing kit (Thermo Fisher Scientific, AM1350). Subsequently, the RNA was purified using phenol/chloroform/isoamyl alcohol (25:24:1), and then the total amount of RNA in each group was detected. Following the pull-down assay, 100 μl of cell lysis, 200 μl of Dynabeads Oligo (dT)25 (Thermo Fisher Scientific, 61005), and 10 μg of RNA in each group were mixed and incubated together for 2 h at 4°C. Subsequently, the samples were washed three times using 500 μl of washing buffer (10 mM of Tris-HCl pH 7.5, 0.15 M of LiCl, 1 mM of EDTA) and then harvested and analyzed using Western blotting. The plasmid containing the antisense of TMEM117 was used as a negative control.

### Statistical Analysis

The statistical analysis was performed using IBM SPSS software (version 24.0), and the data plotting was performed with Prism Graph Pad 6.0 software (33). A value of *p* < 0.05 was considered statistically significant. Unpaired, two-tailed Student’s *t*-tests were used to compare the differences between two groups. One-way ANOVA was used to compare the differences between multiple groups.

## Results

### Identification of a Group of Protein-Coding RNAs Associated With Polycomb Repressive Complex 2-Regulated Genes

**Figure 1:**
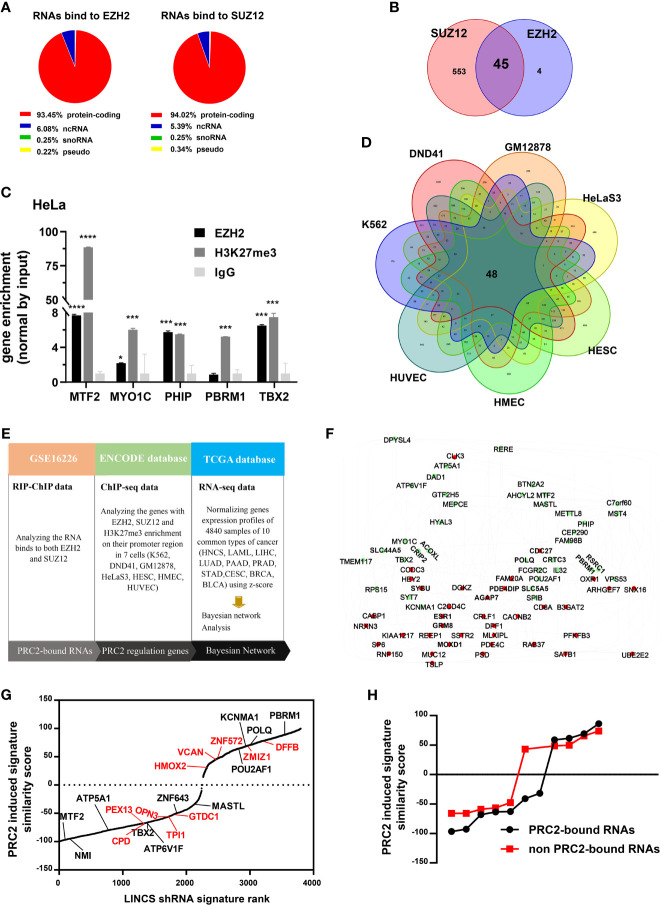
A class of protein coding RNAs are associated with PRC2 complex. **(A)** Analysis of the types of RNAs bound to EZH2 (left) and SUZ12 proteins (right) in fRIP-seq data (GSE67963). **(B)** Forty-five mRNAs bound to both EZH2 and SUZ12 proteins in RIP–ChIP data of HeLa cells (GSE16226). **(C)** RIP assay analyzing the RNAs of MTF2, MYO1C, PHIP, PBRM1, and TBX2, which bind to EZH2 protein and histone H3 with tri-methylation of lysine 27 in HeLa cells, and IgG as negative control (n = 3). **(D)** Genes with EZH2, SUZ12, and H3K27me3 enrichment on their promoter region (−5 kb, +1 kb) in seven cells of the ENCODE database. **(E)** The workflow of Bayesian network analysis of panel **(F)**. **(F)** The Bayesian gene regulatory network of 45 PRC2-bound RNAs and 48 PRC2-regulated genes. Each group of genes is shown by different colors. Green indicates PRC2-bound RNAs, and red indicates PRC2-regulated genes. The arrows between genes represent gene expression regulation. **(G)** The PRC2 induced signature similarity score of some PRC2-bound RNAs and control genes knockout in the LINCS database. The genes in black represent PRC2-bound RNAs, and those in red color indicate control genes with their expression nearest to the PRC2-bound RNAs. **(H)** Comparison of the similarity scores between PRC2-bound RNAs and non-PRC2-bound RNAs of panel **(G)**. Data are represented as means ± SD, *p < 0.05, ***p < 0.001, ****p < 0.0001, unpaired, two-tailed, Student’s *t*-test. PRC2, polycomb repressive complex 2; RIP, RNA immunoprecipitation; ChIP, chromatin immunoprecipitation; LINCS, Library of Integrated Network-Based Cellular Signatures.

To investigate whether protein-coding RNAs epigenetically regulate gene expression in cancer, we first studied whether protein-coding RNAs were physically associated with PRC2 complex. It was pointed out that only EZH2 and SUZ12 proteins could bind RNA with high affinity *in vitro*. Therefore, we only studied the RNAs bound to EZH2 and SUZ12 proteins but not bound to other subunits of PRC2 complex. The fragments of RNAs that bound to EZH2 and SUZ12 proteins were analyzed in the fRIP-seq data of EZH2 and SUZ12 (GSE67963) using the annotatePeak function of ChIPseeker package of R language (34). The results revealed that of the total fragments identified as bound to EZH2 protein, 4.49% were located in the introns and 41.59% were located in exons (**Figure S1A**). Of the fragments of ncRNAs identified as bound to EZH2 protein, 11.47% were located in introns and 31.8% were located in exons (**Figure S1B**). However, of the fragments of protein-coding RNAs identified as bound to EZH2 protein, only 3.79% were located in introns and 42.55% were located in exons (**Figure S1C**). Of the total RNAs with their exons bound to EZH2 or SUZ12 protein, more than 90% were protein-coding RNAs (**Figure 1A**, **Tables S4**, **S5**). The RIP–ChIP data of EZH2 and SUZ12 in HeLa cells (GSE16226) were also analyzed (**Table S6**). The results showed that 45 protein-coding RNAs were bound to both EZH2 and SUZ12 proteins (**Figure 1B**). Subsequently, the RIP assay was exploited to confirm that some of them indeed interacted with EZH2 protein and histone H3 with tri-methylation of lysine 27. The result showed that the RNA of MTF2, MYO1C, PHIP, PBRM1, and TBX2 indeed bound to EZH2 protein and histone H3 with tri-methylation of lysine 27 (**Figure 1C**). Genes with their promoter region enriched with EZH2, SUZ12, and H3K27me3 were considered to be regulated by PRC2-mediated histone H3K27me3 modification. Therefore, we identified PRC2-regulated genes in the ChIP-seq data of EZH2, SUZ12, and H3K27me3 in seven cell lines of ENCODE database (**Table S7**). The result showed that the promoters of 48 genes were occupied by EZH2, SUZ12s and H3K27me3 in all seven cell lines and defined as PRC2-regulated genes (**Figure 1D**). Bayesian network can effectively identify the causal relationship between biological factors (35). Therefore, we studied whether these 45 PRC2-bound transcripts regulated the expression of 48 PRC2-regulated genes in cancer using Bayesian gene regulatory networks (**Figures 1E, F**, **Table S8**). Forty-five genes with their mRNA levels nearest to PRC2-bound transcripts were selected as control genes, and the same analysis was repeated (**Figure S2A**, **Table S9**). In addition, another 45 genes selected randomly by rand function of Excel were also considered as control genes and repeated the same analysis (**Figure S2B**, **Table S9**). In comparison with control genes, PRC2-bound transcripts indeed regulated the expression of PRC2-regulated genes (**Figure 1F**). The LINCS project studies how cells respond to various genetic and environmental stressors. To further confirm our results, 48 PRC2-regulated genes were defined as PRC2-regulated signature, and then the similarity scores between PRC2-bound transcripts and PRC2-regulated signature were evaluated using query tool of LINCS database. Forty-five genes with their mRNA expression level nearest to PRC2-bound transcripts were selected as control genes. In the LINCS project, only 11 PRC2-bound transcripts and 10 control genes were studied. The absolute value of similarity scores of PRC2-bound transcripts were higher than that of control genes, indicating that PRC2-bound transcripts were related to PRC2-regulated signature (**Figures 1G, H**). In conclusion, PRC2-bound transcripts were associated with PRC2-regulated genes.

### Association Between Polycomb Repressive Complex 2-Bound Transcripts and Histone Modification

**Figure 2:**
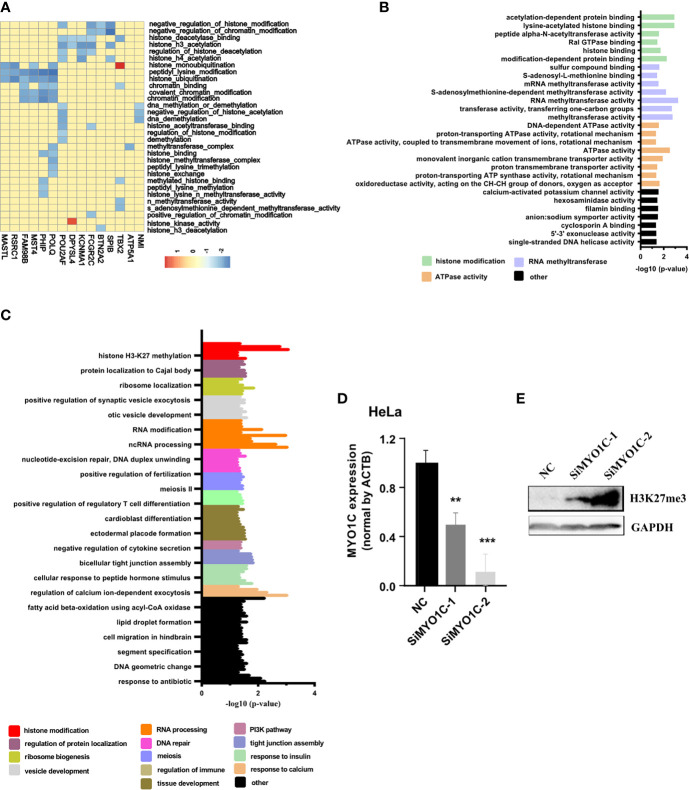
GSEA and GO confirming that PRC2-bound RNAs are associated with histone modification. **(A)** Heatmap showing the PRC2-bound RNAs involved in histone modification in GSEA. The enrichment score (ES) is shown as color intensity. Blue indicates PRC2-bound RNAs negative relevant to histone modification, and the red indicates the opposite. **(B, C)** GO analysis of the enrichment items of PRC2-bound transcripts in sub-ontologies of biological processes **(B)** and molecular function **(C)**. **(D, E)** qRT-PCR detecting the mRNA expression level of MYO1C **(D)** and Western blotting analyzing the modification level of H3K27me3 **(E)** in HeLa cells transfected with SiMYO1Cs for 48 h (negative control (NC)). Data are represented as means ± SD, **p < 0.01, ***p < 0.001, unpaired, two-tailed, Student’s *t*-test. GSEA, gene set enrichment analysis; GO, gene ontology; PRC2, polycomb repressive complex 2.

To further study the functions of PRC2-bound RNAs, we performed the GSEA in the cervical cancer patients of TCGA database (CESC). The histone modification-related pathways were displayed using heatmap. The results revealed more than half of PRC2-bound transcripts markedly enriched on the histone modification-related pathways, indicating these transcripts participated in the regulation of histone modification (**Figure 2A**, **Table S10**). To further confirm our conclusion, the GO analysis was also performed to analyze the items enrichment of PRC2-bound transcripts. The results revealed that histone modification-related items were enrichment in both BP and MF categories (**Figures 2B, C**, **Table S11**). MYO1C, one of the PRC2-bound transcripts, was selected to prove that it regulated the modification level of H3K27me3. We knocked down the expression of MYO1C in HeLa cells using siRNAs and then analyzed the level of H3K27me3 using Western blotting. We found that the level of H3K27me3 markedly increased after 48 h of transfection (**Figures 2D, E**, **Figure S3**).

**Figure 3:**
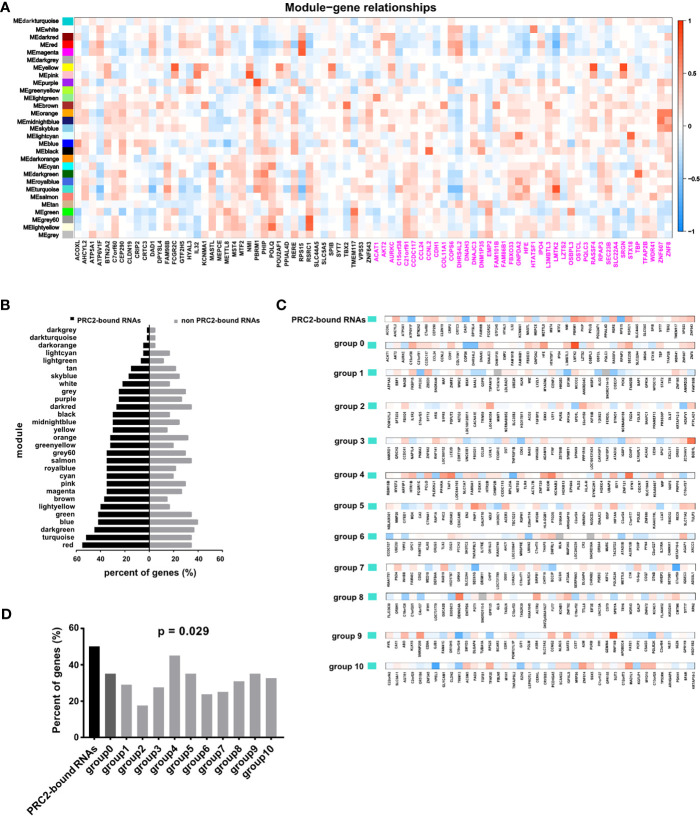
WGCNA confirming PRC2-bound RNAs are associated with histone modification. **(A)** WGCNA analyzing the relationships between modules and PRC2-bound transcripts or control transcripts. The correlation coefficient is shown as color intensity. Red indicates positive correlation, and blue indicates the opposite. The genes with black color represent PRC2-bound transcripts, and genes with magenta color represent control genes with their expression nearest to PRC2-bound transcripts. **(B)** Analyzing the proportion of related genes in the PRC2-bound transcripts or control genes of each module in panel **(A)**. The related gene is defined as the absolute of correlation coefficient greater than 0.2 and *p* < 0.05. **(C)** The relationship between the turquoise module and PRC2 binding RNAs (line 1), or control genes with their expression nearest to PRC2 binding RNAs (group 0), or control genes selected randomly (groups 1–10). The correlation coefficient is shown as color intensity. Red indicates positive correlation, and blue indicates the opposite. **(D)** The proportion of related genes of each group in panel **(C)**. The absolute value of correlation coefficient greater than 0.2, and *p* < 0.05 is indicates related genes. WGCNA, weighted gene co-expression network analysis; PRC2, polycomb repressive complex 2.

In addition, the WGCNA was also performed in CESC to confirm our idea. First, the gene co-expression modules were calculated (**Figure S4**). Subsequently, we analyzed the relationship between co-expression modules and PRC2-bound transcripts, or control genes (**Figure 3A**). The genes with absolute value of correlation coefficient greater than 0.2 and *p* < 0.05 were considered to be related to modules. We next calculated the proportion of related genes in the PRC2-bound transcripts or in the control genes of each module. Among the PRC2-bound transcripts, the two highest modules were red and turquoise modules (**Figure 3B**). GO analysis revealed that genes in red module were associated with RNA processing and those in turquoise module were associated with histone modification and RNA transcription (**Figure S5**, **Table S12**), indicating that PRC2-bound transcripts were related to histone modification and RNA transcription. Next, we focused on the relationship between turquoise module and PRC2-bound transcripts. We randomly selected 10 sets of genes as control genes and analyzed their relationship with turquoise module (**Figure 3C**). The proportion of related genes in PRC2-bound transcripts was the highest (**Figure 3D**), confirming that PRC2-bound transcripts were associated with histone modification.

### TMEM117 Inhibits TSLP Expression Through EZH2-Mediated H3K27me3 Modification

**Figure 4:**
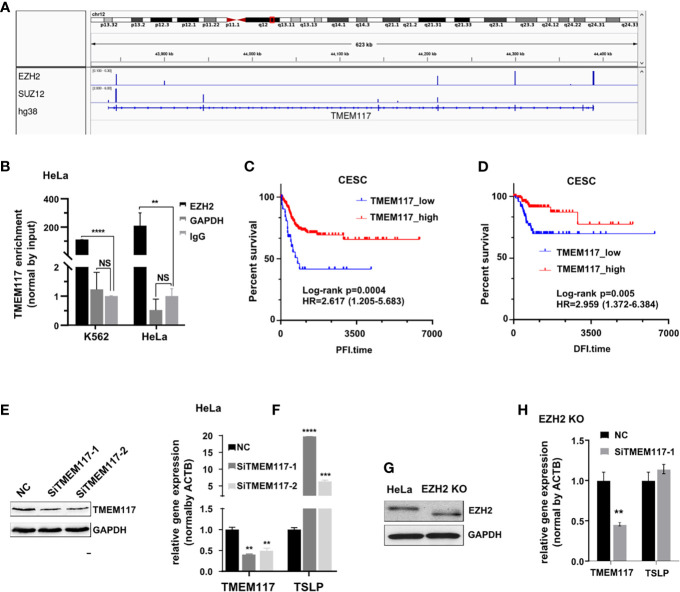
TMEM117 altering TSLP expression. **(A)** Both EZH2 and SUZ12 proteins bind to the exon of TMEM117 in fRIP-seq data (GSE67963). **(B)** RIP assay analyzing the RNA of TMEM117 bound to EZH2 protein in HeLa and K562 cells, with GAPDH and IgG as negative control (n = 3). **(C, D)** Kaplan–Meier survival curves comparing disease-free interval (**C**, N = 176) and progression-free interval (**D**, N = 307) between low and high expression of TMEM117 in CESC. **(E)** Immunoblotting analyzing the TMEM117 level in HeLa cells transfected with siTMEM117s for 48 h. **(F)** qRT-PCR analyzing the mRNA expression of TSLP in HeLa cells transfected with SiTMEM117s for 48 h (n = 3). **(G)** Immunoblotting analyzing the EZH2 level in HeLa cells with EZH2 knockout using CRISPR/Cas9. The gels cropped from different parts of the same gel using clear delineation with white space. We considered that Cas9 shifted the open reading frame (ORF) legion, leading to a truncated EZH2 protein, in which the N-terminus was translated into protein but the C-terminus lacked this domain. Therefore, the band of EZH2 in the knockout cells showed faster migration. **(H)** qRT-PCR analyzing the mRNA expression of TSLP in EZH2 knockout HeLa cells transfected with siTMEM117s for 48 h (n = 3). Data are represented as means ± SD, **p < 0.01, ***p < 0.001, ****p < 0.0001, unpaired, two-tailed, Student’s *t*-test. RIP, RNA immunoprecipitation; CESC, cervical cancer.

We analyzed the RNAs bound on both EZH2 and SUZ12 proteins in the fRIP-seq data (GSE67963). The result showed that both SUZ12 and EZH2 protein bound to the exon of TMEM117 (transmembrane protein 117), a typical protein-coding gene (**Figure 4A**). The RIP assay was also exploited to confirm the interaction between RNA of TMEM117 and EZH2 protein in both K562 and HeLa cells. Interestingly, this interaction in HeLa was approximately twice than that of K562 cells (**Figure 4B**). In addition, the Kaplan–Meier analysis was applied to study the relationship between the expression level of TMEM117 and the prognosis of cancer patients. The result revealed that the expression of TMEM117 was associated with the survival of CESC patients rather than LAML patients of TCGA database (**Figures 4C, D**, **Figure S6**). Therefore, TMEM117 was selected for further study of the HeLa cells. First, we studied whether TMEM117 regulated the expression of PRC2-regulated genes. We knocked down TMEM117 in HeLa cells using siRNAs (**Figure 4E**, **Figure S7**). Subsequently, we detected the expression of TSLP, a gene downstream of TMEM117 calculated by the Bayesian gene regulatory network. The result revealed that TSLP significantly increased after 48 h of siTMEM117s transfection (**Figure 4F**). We also detected whether TMEM117 altered the expression of TSLP in the HeLa cells with EZH2 knockout (**Figure 4G**, **Figure S8**). Nevertheless, knockdown of TMEM117 did not affect the expression TSLP in the HeLa cells with EZH2 knockout (**Figure 4H**), suggesting that TMEM117-regulated TSLP expression was EZH2 dependent.

**Figure 5:**
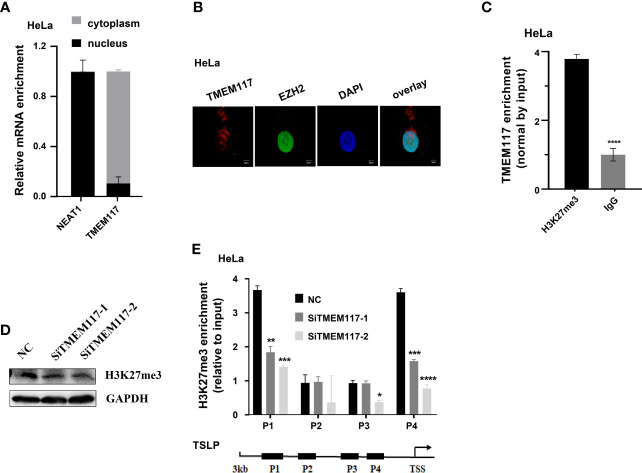
TMEM117 altering TSLP expression dependent on EZH2-mediated modification of H3K27me3. **(A)** The distribution of TMEM117 RNA in cytoplasm and nucleus, with NEAT1 as control (n = 3). **(B)** RNA-FISH and immunofluorescence staining RNA of TMEM117 (red) and EZH2 protein (green) in HeLa cells. **(C)** RIP assay analyzing the interaction between RNA of TMEM117 and histone 3 with tri-methylation on lysine 27, with IgG as negative control (n = 3). **(D)** Immunoblotting analyzing the modification level of H3K27me3 in HeLa cells transfected with siTMEM117s for 48 h. **(E)** ChIP analyzing enrichment of H3K27me3 on the promoter region of TSLP in HeLa cells transfected with siTMEM117s for 48 h (n = 3). Data are represented as means ± SD, *p < 0.05, **p < 0.01, ***p < 0.001, ****p < 0.0001, unpaired, two-tailed, Student’s *t*-test. FISH, fluorescence *in situ* hybridization; RIP, RNA immunoprecipitation; ChIP, chromatin immunoprecipitation.

Therefore, we asked whether TMEM117 suppressed TSLP expression through EZH2-mediated H3K27me3 modification. Because the protein of EZH2 located in nucleus and the modification of histone happened in the nucleus, we first analyzed whether the RNA of TMEM117 occurred in the nucleus using cell fractions analysis and FISH assay. The result revealed that part of the RNA of TMEM117 is localized in the nucleus (**Figures 5A, B**). The RIP assay was also performed to demonstrate that the RNA of TMEM117 interacted with histone 3 with tri-methylation of lysine 27 (**Figure 5C**). We next studied whether TMEM117 affected the modification level of H3K27me3. We knocked down TMEM117 in HeLa cells using siRNAs and then detected the modification level of H3K27me3. The result revealed that TMEM117 knockdown did not affect the modification level of H3K27me3 (**Figure 5D**). However, the ChIP assay proved that TMEM117 silencing dramatically reduced the occupation of H3K27me3 on the promoter region of TSLP (**Figure 5E**). In conclusion, TMEM117 regulated TSLP expression through EZH2-mediated H3K27me3 modification.

### RNA Recognition Motif Contributes to Protein-Coding RNA Binding to Polycomb Repressive Complex 2

**Figure 6:**
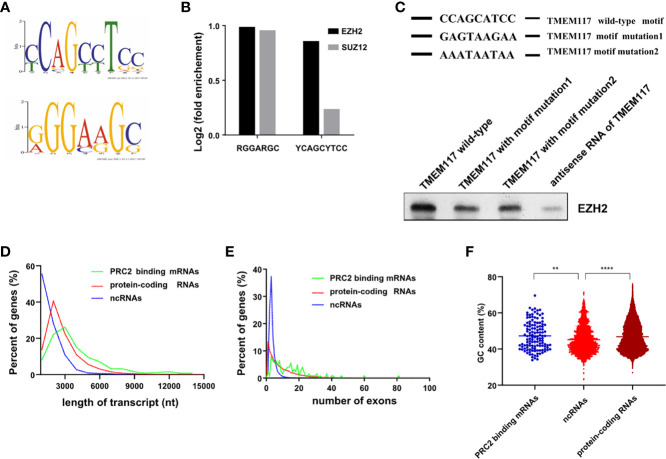
Characteristics of PRC2-bound RNAs. **(A)** Analysis of the RNA recognition motif of PRC2 using the MEME algorithm in fRIP-seq data (GSE67963). **(B)** Analysis of the enrichment of two RNA recognition motifs in fRIP-seq data of EZH2 and SUZ2 (GSE67963). **(C)** The RNA-pulldown assay analyzing the interaction between EZH2 protein and the RNA of TMEM117 with or without the RNA recognition motif mutation. The antisense of TMEM117 is used as the negative control. The gels cropped from different parts of the same gel using clear delineation with white space. **(D–F)** Analysis of the length of RNAs **(D)**, number of exons **(E)**, and GC content **(F)** in each group of transcripts. **p < 0.01, ****p < 0.0001, unpaired, two-tailed, Student’s *t*-test. The GC content is analyzed by Mann–Whitney test. PRC2, polycomb repressive complex 2; GC, guanine–cytosine.

Previous studies indicated that RNAs with some characteristic sequences help them bind to proteins more easily. Therefore, we analyzed the RNA recognition motif of PRC2 complex in the fRIP-seq data using the MEME algorithm. The result revealed there were two RNA recognition motifs of PRC2 complex (**Figure 6A**). We next quantified the association between PRC2 complex and these two motifs. The result revealed that both of them were associated with EZH2 and SUZ12 proteins (**Figure 6B**). A G-rich motif was identified, consistent with previous reports (15, 16). Subsequently, the YCAGCYTCC motif was pinpointed and focused on. To study the function of YCAGCYTCC motif, we performed the RNA-pulldown assay to study the interaction between EZH2 protein and RNA of TMEM117 with or without the YCAGCYTCC motif mutation. The results revealed that RNA of TMEM117 with YCAGCYTCC motif mutation dramatically decreased its interaction with EZH2 protein (**Figure 6C**, **Figure S9**), indicating that this motif contributed to their interaction. We next compared the characteristics between PRC2-bound transcripts and protein-coding RNAs, such as length, the number of exons, and the guanine–cytosine (GC) content of transcripts. The result showed there was no difference among them (**Figures 6D–F**).

### Identification of a Class of Protein-Coding RNAs Associated With H3K27me3 in Mice

**Figure 7:**
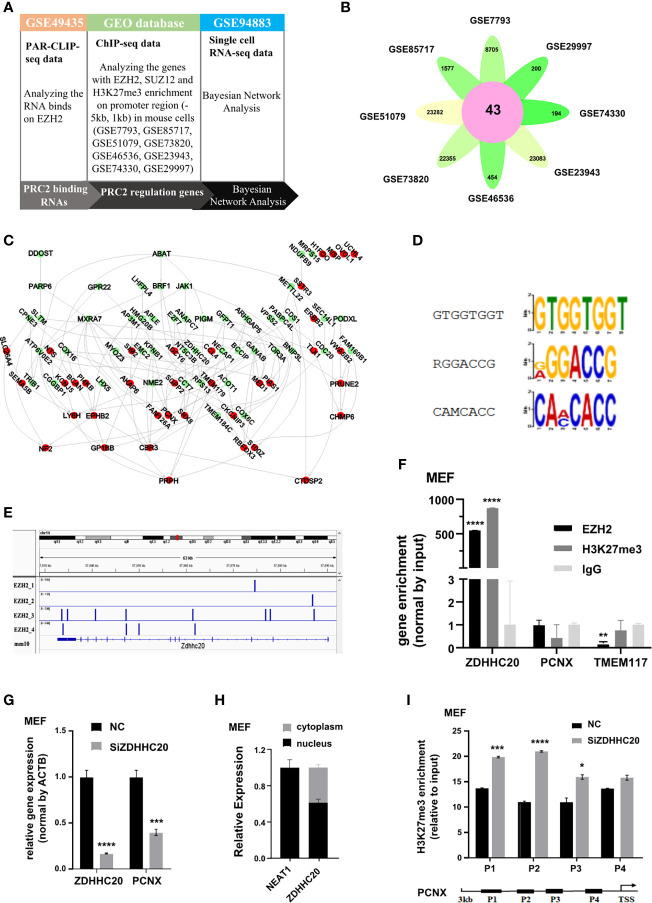
PRC2 binding RNAs regulate histone methylation in mouse. **(A)** The workflow of Bayesian network analysis of panel **(B)**. **(B)** Genes with EZH2, SUZ12, and H3K27me3 enrichment on their promoter region (−5 kb, +1 kb) in eight ChIP-seq data. **(C)** The Bayesian gene regulatory network analyzing the regulatory relationship between PRC2-bound RNAs and PRC2-regulated genes in mouse. **(D)** Analysis of the enrichment of three RNA recognition motifs of EZH2 protein in par-CLIP-seq data of EZH2 (GSE49435) using MEME algorithm. **(E)** Four repeats of EZH2 par-CLIP-seq data (GSE67963) reveal that EZH2 binds to the RNA of ZDHHC20 in mice. **(F)** RIP assay analyzing the RNA of ZDHHC20 binding to EZH2 protein and histone H3 with tri-methylation of lysine 27 in MEF cells, with TMEM117, PCNX, and IgG as negative control (n = 3). **(G)** qRT-PCR detecting the mRNA expression of PCNX in MEF cells transfected with siZDHHC20 for 48 h (n = 3). **(H)** The distribution of ZDHHC20 in the cytoplasm and nucleus, with NEAT1 is used as a control (n = 3). **(I)** ChIP assay analyzing the enrichment of H3K27me3 on the PCNX promoter in MEF cells transfected with siZDHHC20 for 48 h (n = 3). Data are represented as means ± SD, *p < 0.05, ***p < 0.001, ****p < 0.0001, unpaired, two-tailed, Student’s *t*-test. PRC2, polycomb repressive complex 2; RIP, RNA immunoprecipitation; ChIP, chromatin immunoprecipitation.

PRC2 complex is highly conserved between plants and animals. In humans and mice, PRC2 complex binds to RNA in a similar manner *in vitro* (17). Therefore, we investigated whether the protein-coding RNAs bound to PRC2 complex and regulated histone methylation in mice using Bayesian gene regulatory network analysis (**Figure 7A**). The PRC2-bound RNAs were identified from par-CLIP-seq data of EZH2 protein (GSE49435). Subsequently, genes with their promoter region enriched with EZH2, SUZ12, and H3K27me3 were considered to be PRC2-regulated genes and identified in the eight datasets of ChIP-seq data (**Figure 7B**, **Table S13**). The Bayesian gene regulatory network analysis was performed to study their causal relationship. The results revealed that PRC2-bound RNAs regulated the expression of PRC2-regulated genes (**Figure 7C**, **Table S14**). Subsequently, the RNA recognition motifs of EZH2 protein in mice were analyzed in the par-CLIP-seq data (GSE49435) using the MEME algorithm. Three motifs were identified, and one of them was considered as G-rich motif (**Figure 7D**), consistent with a previous report that EZH2 protein preferentially bound to G-rich RNA was conserved (16). Analyzing four repeats of par-CLIP-seq data of EZH2 (GSE49435) revealed that EZH2 protein directly bound to the RNA of ZDHHC20 (**Figure 7E**). We also performed the RIP assay to confirm that the RNA of ZDHHC20 indeed bound to EZH2 protein and H3 with tri-methylation on lysine 27 in MEF cells (**Figure 7F**). Therefore, ZDHHC20 was selected for further study. We asked whether ZDHHC20 regulated the expression of PRC2-regulated genes. Therefore, we knocked down the expression of ZDHHC20 in MEF cells using siZDHHC20 and then detected the expression of PCNX, a gene downstream of ZDHHC20 calculated by Bayesian gene regulatory network. The result revealed that knockdown of ZDHHC20 in MEF cells significantly decreased the expression of PCNX (**Figure 7G**). We next analyzed the RNA location of ZDHHC20 using the cell fraction analysis. The result revealed that part of the RNA of ZDHHC20 localized in the nucleus, indicating that it had the opportunity to bind to EZH2 protein and mediate H3K27me3 modification (**Figure 7H**). To further prove this, we knocked down ZDHHC20 in MEF cells using siRNA and studied whether it altered the occupation of H3K27me3 modification using ChIP assay. The result revealed that ZDHHC20 knockdown significantly increased the occupation of H3K27me3 on the promoter of PCNX (**Figure 7I**), indicating that ZDHHC20 might regulate PCNX expression through altering the level of H3K27me3 modification.

## Discussion

**Figure 8:**
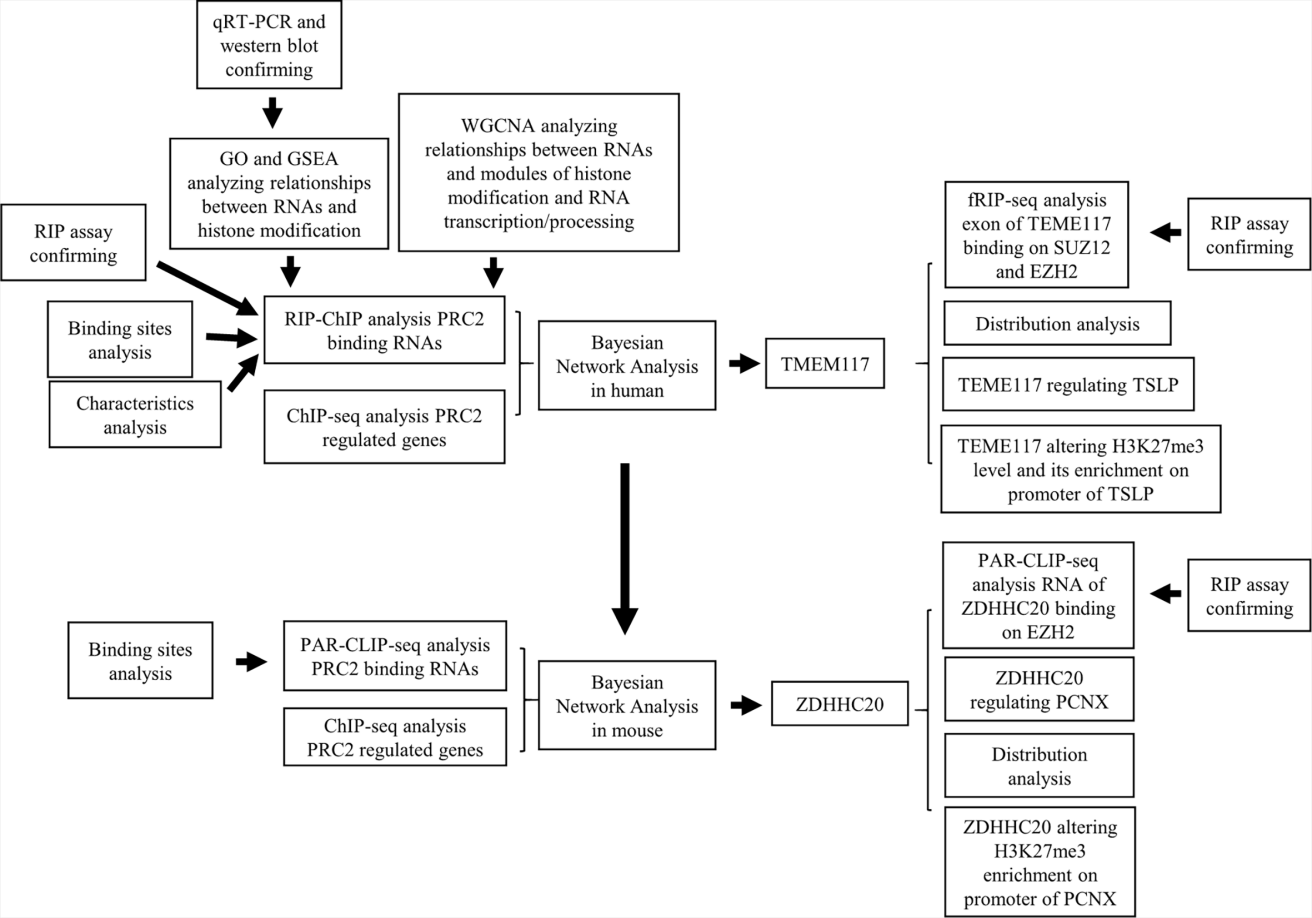
The workflow of this research.

Previous works had revealed that RNA-mediated regulation of PRC2 complex in general. Our data mining also comprehensively analyzed that a class of protein-coding RNAs could bind to PRC2 complex directly. We further found that this class of RNAs regulated the expression of PRC2-regulated genes. TMEM117 is a typical protein-coding gene that contains PRC2 binding motif. We found that the RNA of TMEM117 could bind to PRC2 complex and regulate TSLP expression in an EZH2-dependent manner. We proposed the RNA recognition motif of PRC2 complex might help them bind to the PRC2 complex more easily. Universally, the same regulatory pattern also occurred in mice (**Figure 8**). Combining with previous research, our results support a newly described idea that a class of protein-coding RNAs could bind to PRC2 complex and epigenetically regulate the expression of genes. In this model, like lncRNAs, this class of protein-coding RNAs bound to EZH2 and SUZ12 proteins directly, altering H3K27me3 methylation and epigenetically regulating gene expression. Previous studies pointed out that lncRNAs could serve as a guide or decoy to affect the recruitment of PRC2 complex to specific genomic loci (36). Whether the PRC2 bound RNAs using the same mechanism requires further research.

Subunits of the PRC2 complex, including EZH2 and SUZ12 proteins, have demonstrated high affinity to RNA both *in vivo* and *in vitro* (37). Previous studies and our data mining show that more than 90% of RNAs that bind to EZH2 protein are protein-coding RNA (18, 19). The RNA-pulldown assay also confirmed protein-coding RNAs bound to EZH2 protein, a subunit of PRC2 complex. Except lncRNAs, there are few studies that show the functions of protein-coding RNAs that bind to PRC2 complex. In addition, little is known about the mechanism of interaction between protein-coding RNAs and PRC2 complex. It is well accepted that PRC2–RNA binding can mediate the recruitment of PRC2 complex to the specific chromosome loci (38). For this reason, we hypothesize that these PRC2-bound RNAs may be capable of regulating histone methylation through PRC2 complex. Nowadays, mRNAs are widely accepted to move outside the nucleus and serve as messenger between DNA and protein. Even though there are no studies showing that protein-coding RNAs regulate histone modification, it is well demonstrated that some non-coding RNAs can encode functional proteins (39–41). In this respect, the boundary between non-coding RNAs and protein-coding RNAs is a little arbitrary. Our results considered that, like some bifunctional ncRNAs, protein-coding RNA could also have double functions, regulating histone modification and gene epigenetic expression.

The mechanism of recruitment for PRC2 complex to specific chromosome loci is not very well understood. Several models have been proposed to explain RNA-mediated PRC2 complex recruitment. The “junk mail” model considers that RNAs bound to PRC2 complex are promiscuous. Under this model, RNAs bind to PRC2 complex and serve as a decoy at active genes and as guide to the genes requiring silencing (18). Apart from the “junk mail” model, the masking model shows that RNAs that are not bound to other proteins have a higher chance of binding to PRC2 complex. The RNA-chromatin antagonistic model suggests the competitive binding of RNA and chromatin to PRC2 complex (42). What model the protein-coding RNAs use to mediate the recruitment of PRC2 complex requires further study. Although the consensus motif is not obvious, previous studies show that PRC2 has a strong tendency to bind to the two-hairpin and G-rich RNA. Consistently, we found two RNA recognition motifs for PRC2 complex, including a G-rich motif. The motif mutation decreased the interaction between RNA of TMEM117 and EZH2 proteins, indicating that RNA recognition motif containing may bind to PRC2 complex more readily.

## Conclusion

In summary, we have described a new idea that there is a class of protein-coding RNAs that binds to PRC2 complex and epigenetically regulate gene expression. Our findings help further understand the mechanism of PRC2 complex recruitment and open the search for new functions of protein-coding RNAs.

## Data Availability Statement

The original contributions presented in the study are included in the article/**Supplementary Material**. Further inquiries can be directed to the corresponding authors.

## Author Contributions

ML designed and conducted the experiments, analyzed the data, and wrote and revised the manuscript. XS detected MYO1C-regulated H3K27me3 modification. SG carefully revised the manuscript. YZ designed the experiments and supervised the project. All authors contributed to the article and approved the submitted version.

## Funding

This work was supported by the Youth Program of National Natural Science Foundation of China (31900540) and the international cooperation fund of Shenzhen (GJHZ20180929162002061).

## Conflict of Interest

The authors declare that the research was conducted in the absence of any commercial or financial relationships that could be construed as a potential conflict of interest.

## Publisher’s Note

All claims expressed in this article are solely those of the authors and do not necessarily represent those of their affiliated organizations, or those of the publisher, the editors and the reviewers. Any product that may be evaluated in this article, or claim that may be made by its manufacturer, is not guaranteed or endorsed by the publisher.
