# Supplementary material for: A Class of Protein-Coding RNAs Binds to Polycomb Repressive Complex 2 and Alters Histone Methylation
Source: Front Oncol. 2021 Nov 5;11:739830. doi: 10.3389/fonc.2021.739830 (PMC8602814; doi:10.3389/fonc.2021.739830)
Supplement: Supplementary file 1 [file DataSheet_1.pdf]

A Class of Protein Coding RNAs Bind to PRC2 and Alter Histone Methylation

Meijian Liao[1,2,3*], Xiaolin Sun[1], Shoucui Gao[1], Yaou Zhang[2,4,5*]

[1]Department of Pathology, Xuzhou Medical University, Xuzhou 221004, P.R. China;
[2]State Key Laboratory of Chemical Oncogenomics, Graduate School at Shenzhen, Tsinghua University, Shenzhen 518055, P.R. China;
[3]School of Life Sciences, Tsinghua University, Beijing 100084, P.R. China
[4]Key Lab in Healthy Science and Technology, Division of Life Science, Graduate School at Shenzhen, Tsinghua University, Shenzhen 518055, P.R. China;
[5]Open FIESTA Center, Tsinghua University, Shenzhen 518055, P.R. China.

*Correspondence: zhangyo@sz.tsinghua.edu.cn (Yaou Zhang)
13751153850@163.com (Meijian Liao)

Supplementary Method

The R scripts of Bayesian gene regulatory network

```
library(pcalg)
library(Rgraphviz)
a<-read.csv('expression.csv', header=T)
b<-list(C=cor(a[,1:ncol(a)]), n=nrow(a))
d<-pc(b,indepTest=gaussCItest, alpha=0.05, labels=names(a[1:ncol(a)]), verbose=TRUE)
plot(d)
red<-rep('red',ncol(a))
names(red)<-rep('green',ncol(a))
names(red) <- c('KCNJ5', 'H1FOO', 'TLX1', 'NTS', 'SLC26A4', 'CCL4', 'SGPP2', 'SIM2', 'PRUNE2',
'AKAP6', 'KCNIP3', 'MISP', 'S100Z', 'VWA5B2', 'OVOL1', 'PIAS1', 'BCAN', 'PI4KB', 'ERBB2',
'SEMA5B', 'FAM126A', 'EPHB2', 'PCNX', 'UCHL4', 'TMEM179', 'SNX8', 'NF2', 'ADCY6',
'MED1', 'GP1BB', 'SSTR3', 'LY6H', 'CBR3', 'RBFOX3', 'PRPH', 'CHMP6', 'CTDSP2')    //*PRC2
regulated genes in mouse*//
names(red) <- c('AGAP7', 'ARHGEF7', 'B3GAT2', 'C2CD4C', 'CABP1', 'CACNB2', 'CCDC3',
'CD8A', 'CDC27', 'CLK3', 'CRLF1', 'DGKZ', 'DPF1', 'ESR1', 'FAM20A', 'GRM8', 'HEY2',
'KIAA1217', 'MLXIPL', 'MOXD1', 'MUC12', 'NRXN3', 'OXR1', 'PDE4C', 'PDE4DIP', 'PFKFB3',
'PSD', 'RAB37', 'REEP1', 'RNF150', 'SATB1', 'SNX16', 'SP6', 'SSTR2', 'SYBU', 'TSLP', 'UBE2E2')
//*PRC2 regulated genes in human*//
nAttrs<-list()
nAttrs$fillcolor <- red
plot(d@graph, attrs=list(node=list(label="foo",
fillcolor="lightgreen",fontsize=65,color="white"),edge=list(color="grey")),
nodeAttrs=nAttrs,lwd=0.001)
```

Supplementary Results

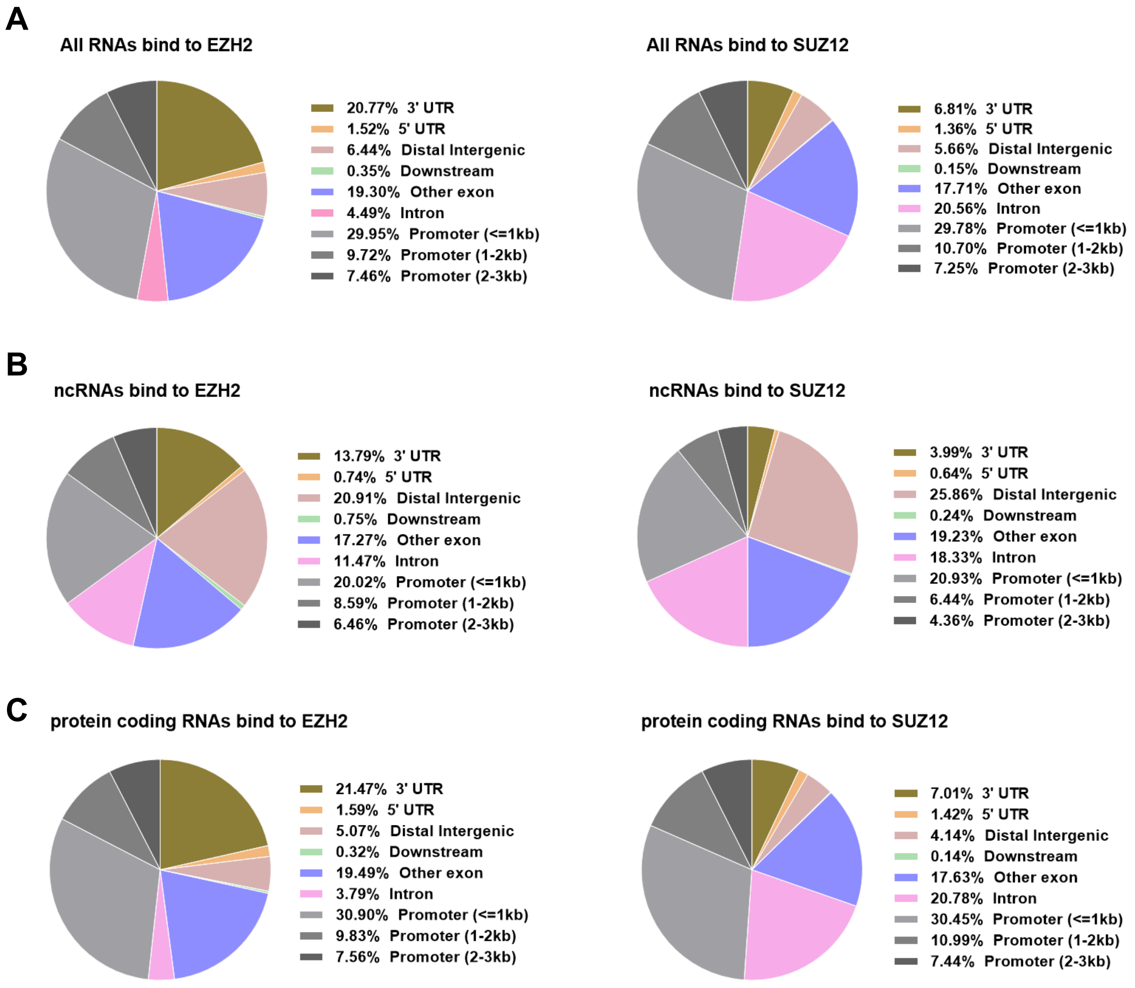

Supplementary Figure 1 : Analysis of the distribution of total RNAs (**A**), ncRNAs (**B**), and protein coding RNAs (**C**) that bound to EZH2 (left) and SUZ12 (right) in fRIP-seq data (GSE67963).

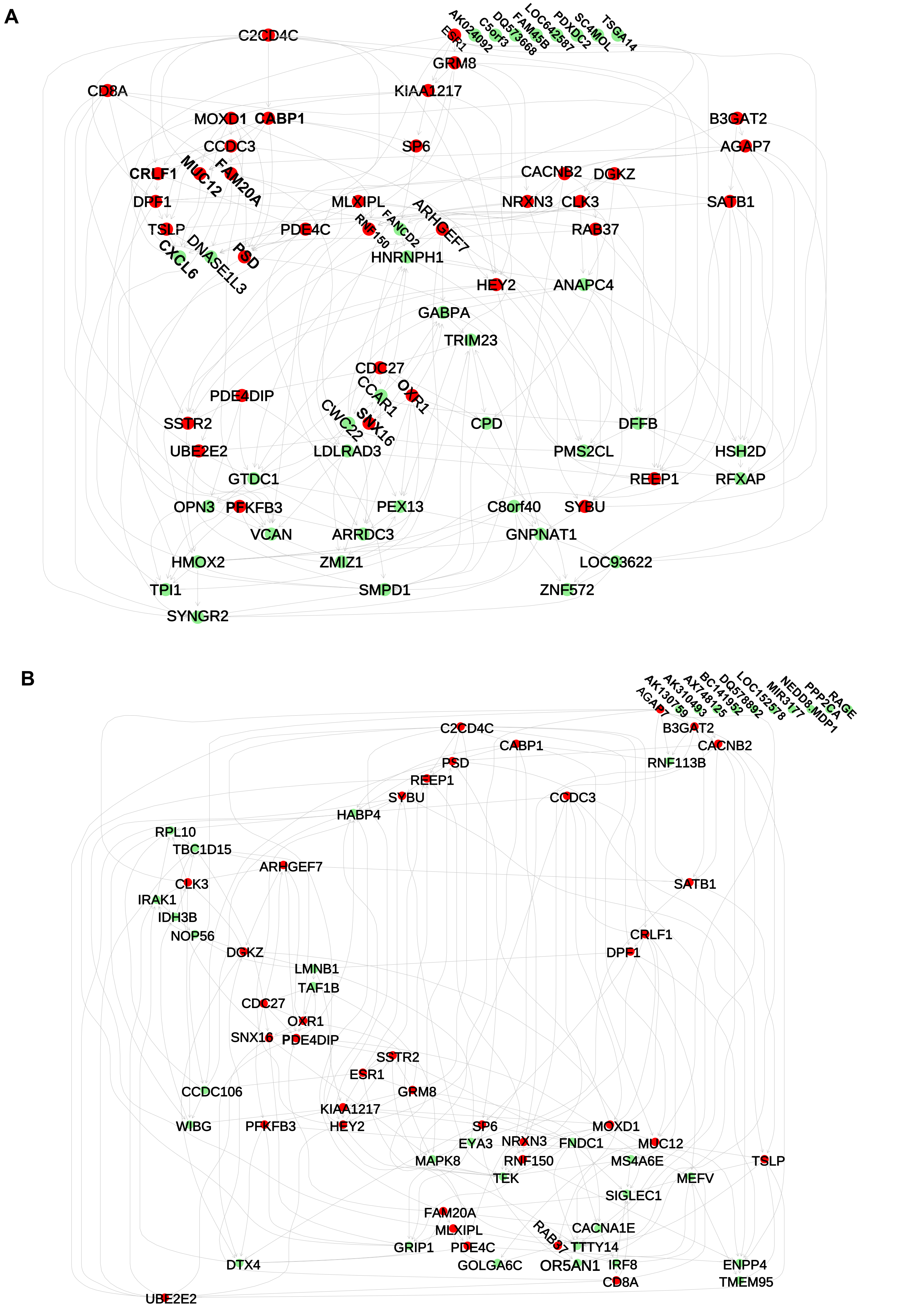

Supplementary Figure 2 : The Bayesian gene regulatory network of control genes and PRC2 induced genes. **(A)** Control genes with their expression nearest to PRC2-bound transcripts. **(B)** Control genes selected randomly using the rand function of Excel.

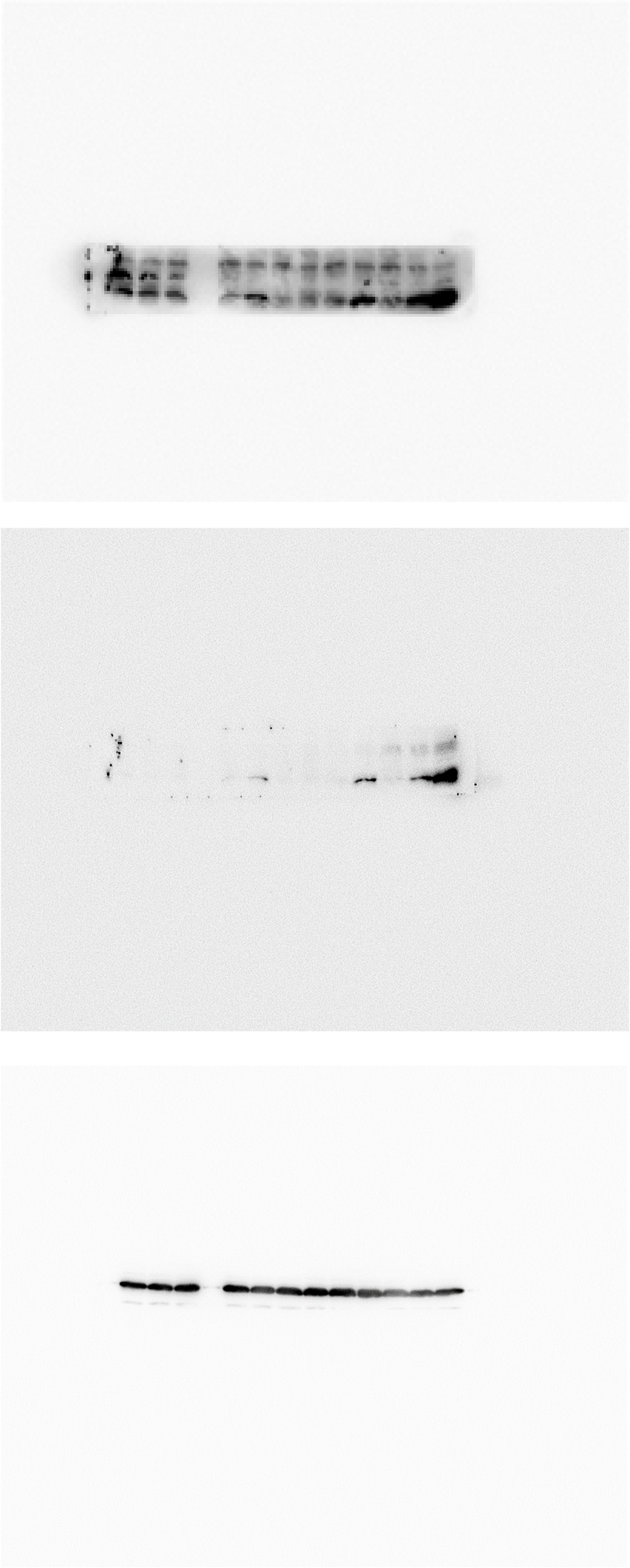

Supplementary Figure 3 : The full-length gels of Figure 2E and Figure 5D.

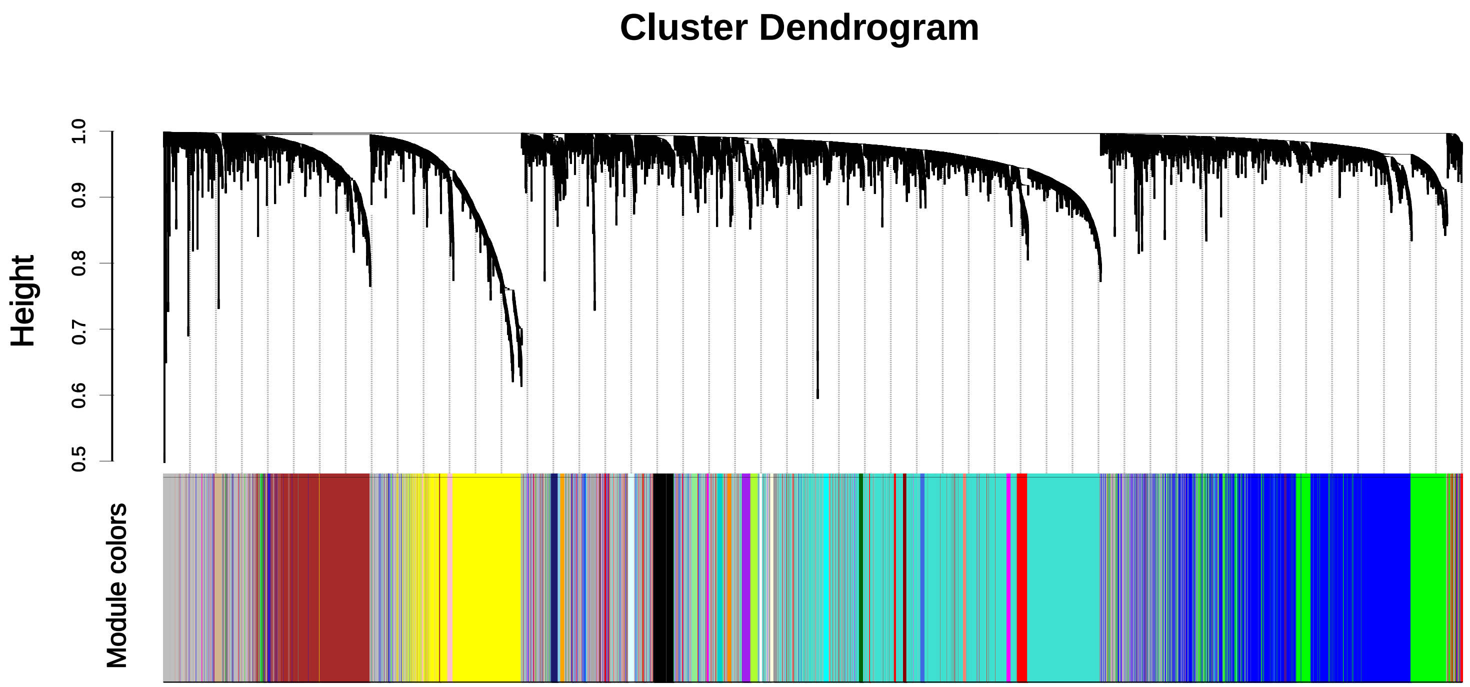

Supplementary Figure 4 : WGCNA analysis the gene co-expression modules of TCGA cervical cancer.

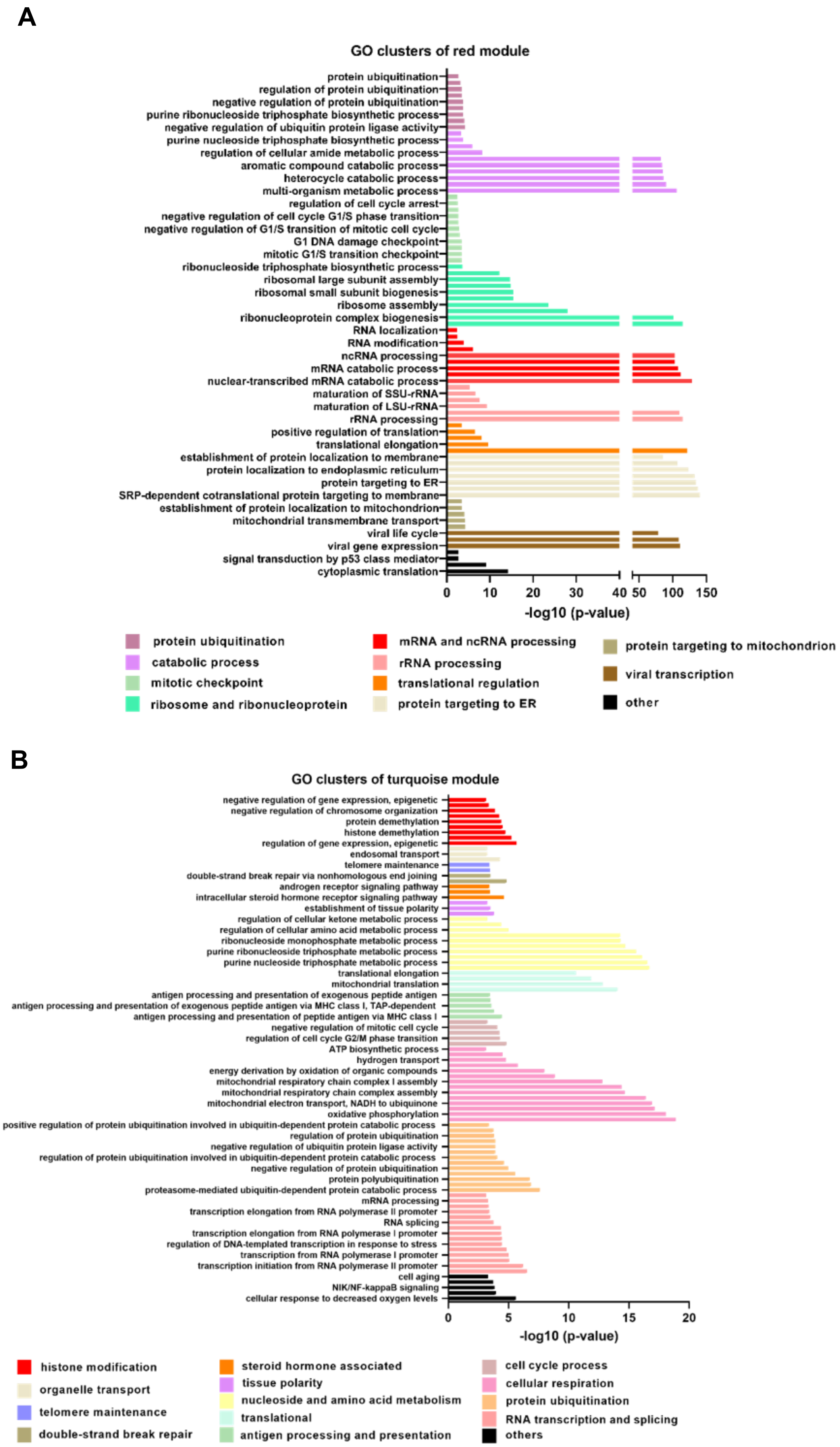

Supplementary Figure 5 : Gene ontology (GO) analysis functions of genes enrichment in red **(A)** and turquoise **(B)** modules.

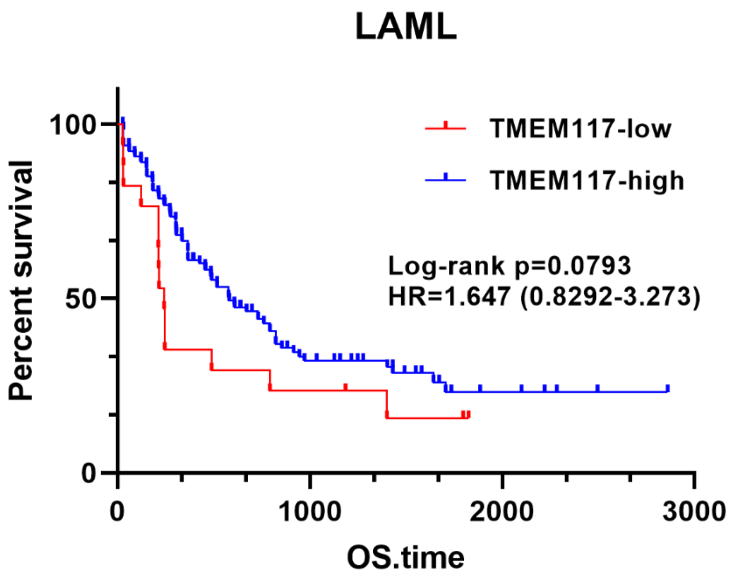

Supplementary Figure 6 : Kaplan-Meier survival curves comparing overall survival between low- and high-expression of TMEM117 in acute myeloid leukemia (LAML).

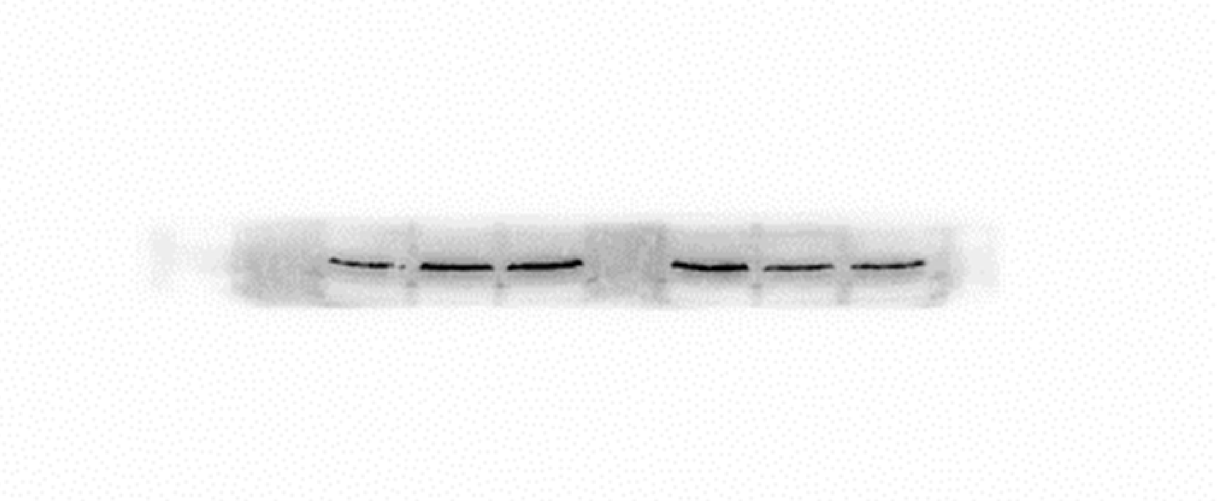

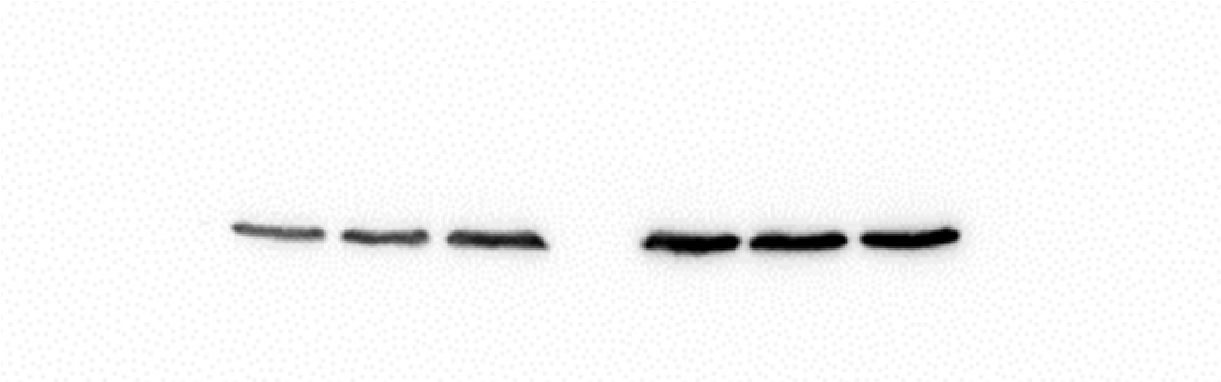

Supplementary Figure 7 : The full-length gels of Figure 4E.

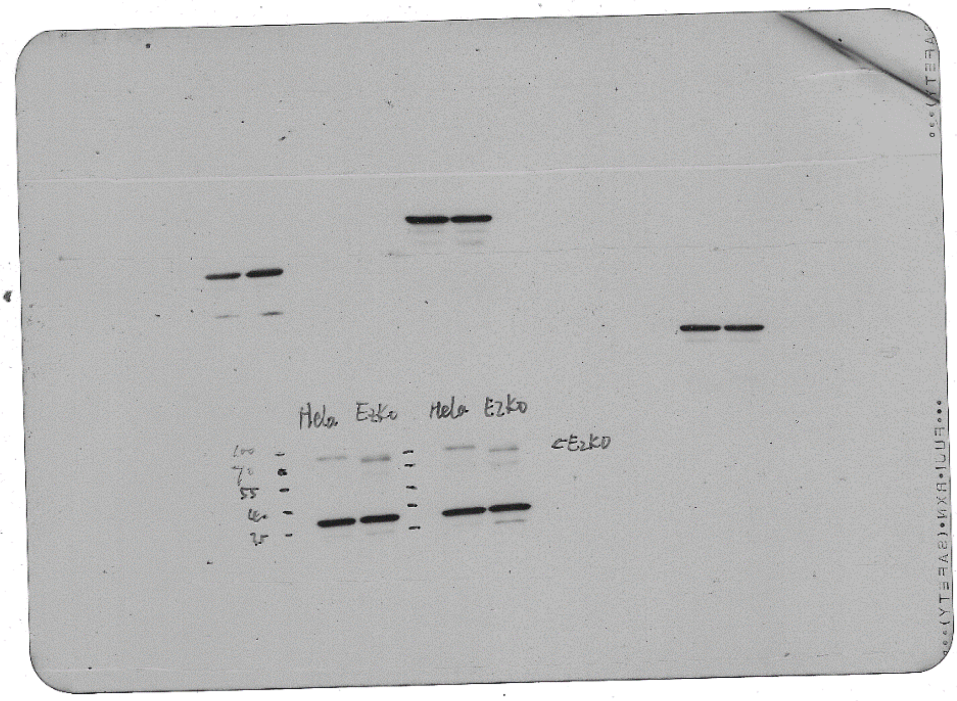

Supplementary Figure 8 : The full-length gels of Figure 5F.

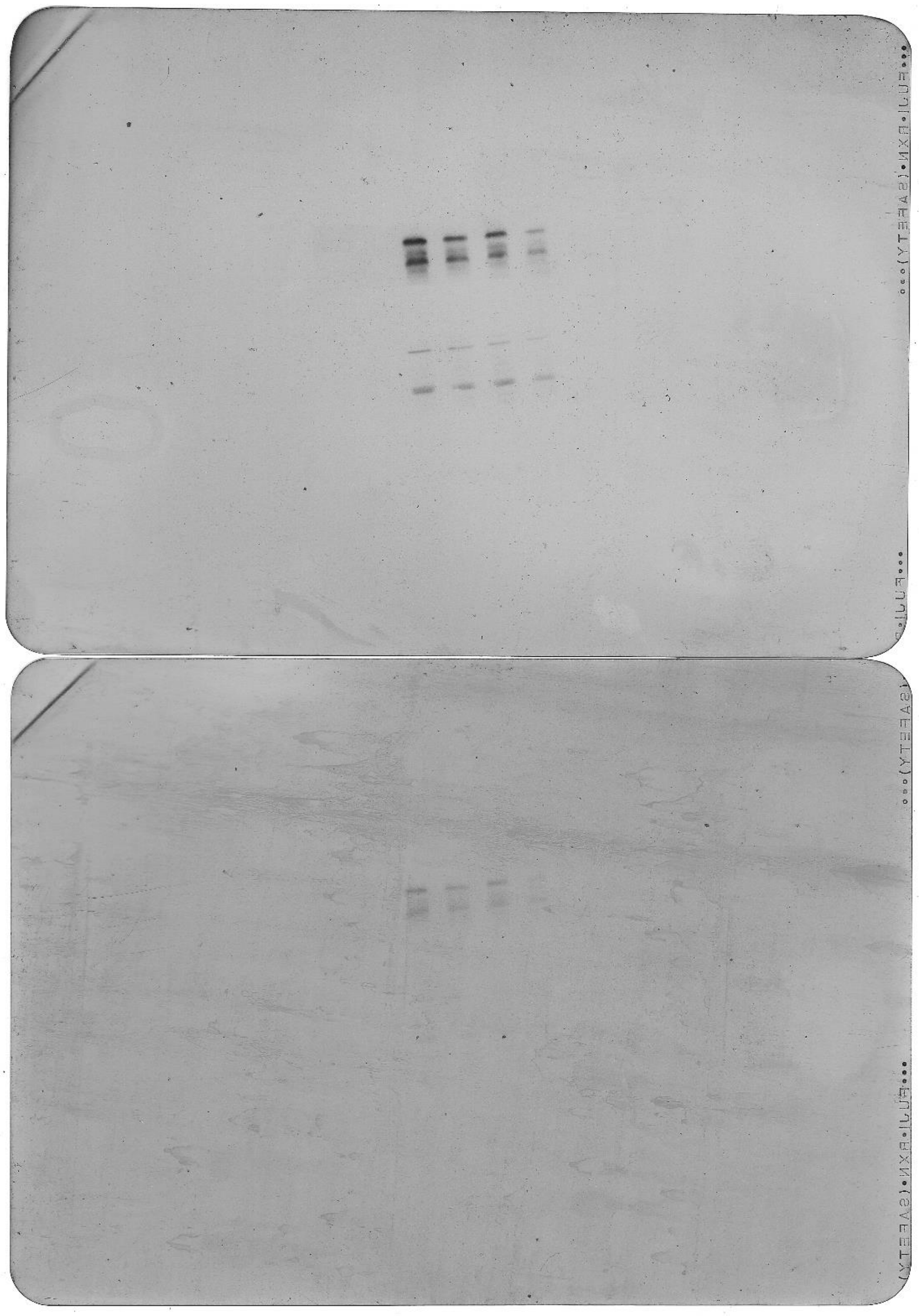

Supplementary Figure 9 : The full-length gels of Figure 6C.

Supplementary Tables

Table S1. The sequences of SiRNAs in this study.

SiRNAs	Sense (5'-3')	Antisense (5'-3')
SiTMEM117_1	CGAAUCUACUAGUGCAACA	UGUUGCACUAGUAGAUUCG
SiTMEM117_2	AAAGCAACCUUGAGUGUAA	UUACACUCAAGGUUGCUUU
SiMYO1C_1	AUACGAAGCUUUCCUGCAA	UUGCAGGAAAGCUUCGUAU
SiMYO1C_2	AGAACAUGGUGUGGAAAUA	UAUUUCCACACCAUGUUCU

Table S2. The detailed information of datasets in this study.

Deposited Data	Source	Identifier
TCGA data	UCSC Xena (version 2017-10-13)	http://xena.ucsc.edu/
ChIP-seq in human cells; Distribution of RNAs in human cells	ENCODE	https://www.encodeproject.org/
fRIP-seq in human cells	Hendrickson DG et al., 2016 [1]	GSE67963
RIP-ChIP in human cells	Khalil AM et al., 2009 [2]	GSE16226
	Juan AH et al., 2016 [3]	GSE85717
	Tumes DJ et al., 2013 [4]	GSE51079
	Onodera A et al., 2015 [5]	GSE73820
ChIP-seq in mouse cells	Mozzetta C et al., 2014 [6]	GSE46536
	Marks H et al., 2012 [7]	GSE23943
	Kloet SL et al., 2016 [8]	GSE74330
	He A et al., 2012 [9]	GSE29997
	Ishak CA et al., 2016 [10]	GSE77993
PAR-CLIP-seq in mouse	Kaneko S et al., 2013 [11]	GSE49435
Gene expression in mouse	Rizvi AH et al., 2017 [12]	GSE94883

Table S3. The primers of genes in this study.

GENE	Forward primer	Reverse primer
Has-ACTB	TGACGTGGACATCCGCAAAG	CTGGAAGGTGGACAGCGAGG
Has-NETA1	ACATTGTACACAGCGAGGCA	CATTTGCCTTTGGGGTCAGC
Clone-Has-TMEM117	CGGGGTACCGAGAAGCCATGCCTCCAGCT	CTAGTCTAGATCACTGCTGTATAATTCTTATTTATT
Clone_TMEM117_motif_mution	GAGTAAGAACTGGTCTCGCAT	AAATAGTAACGAAAGTCTTTACC
Has-TMEM117	TCCCTGGTCTCGCATGATTG	GCAAGTAGCCATAGAAGCACCTT
Has-TSLP-promoter1	TCCTTTGCTCCATTCTC	ATCCTGTAAGCCGTATCT
Has-TSLP-promoter2	ACCCTGCCTACCTCATC	AGAACCTTCGGCTTCAT
Has-TSLP-promoter3	AGCCGAAGGTTCTAGTGG	AAGGAAAGGGCATGGTG
Has-TSLP-promoter4	AGCGGTGAATCAGAGGT	GGAGAAAGGAGGTTGGAC
Has-TSLP	GACCTGACCCAAGCTCTTA	TCCTTTCTCCCTAATCCTC
Mmu-ACTB	GGCTGTATTCCCCTCCATCG	CCAGTTGGTAACAATGCCATGT
Mmu-NETA1	AGATACACTCCGCAGAAAC	CCTAACACCGAAAATAACTAA
Mmu-PCNX	CCTGCCCTTCACGCTTTACAT	GCTCTGTGGAGTCGATAGTTGAC
Mmu-ZDHHC20	GGAAAGACCGTTGTTTACCTTGT	ACTCCTTCTCATAACGCTCCTTC
Mmu-PNCX-promoter1	AGGGATTGACACCTAATAGAC	CACGCTGCCAAACCACCA
Mmu-PNCX-promoter2	AGGGTGAAAGAGGCAGTT	TGGATACAGTATGTTGAGGC
Mmu-PNCX-promoter3	CGGATGAGTCCAGTGCTA	GGAAGATGTAAACCAGGATAAA
Mmu-PNCX-promoter4	TCCTGGTTTACATCTTCCTT	ATAGTGACCTAATGGCTTGA

Table legends

Table S4. Analyzing the SUZ12-bound transcripts in fRIP-seq data (GSE67963).

Table S5. Analyzing the EZH2-bound transcripts in fRIP-seq data (GSE67963).

Table S6. Analyzing the RNAs binding to both EZH2 and SUZ12 in HeLa cell (GSE16226).

Table S7. Analyzing genes with their promoter region enriched with EZH2, SUZ12 and H3K27me3 in ChIP-seq data (ENCODE).

Table S8. The gene expression profile data used in Bayesian gene regulatory network analysis of human.

Table S9. The expression profile of control genes used in Bayesian gene regulatory network analysis of human.

Table S10. GSEA analysis the pathways enrichment on PRC2 binding RNAs.

Table S11. GO analysis the items enrichment of PRC2-bound transcripts.

Table S12. GO analysis the pathways enrichment of each gene co-expression module.

Table S13. Analyzing genes with their promoter region enriched with EZH2, SUZ12 and H3K27me3 in mouse.

Table S14. The gene expression profile data used in Bayesian gene regulatory network analysis of mouse.

References

1. D GH, Kelley DR, Tenen D, Bernstein B, Rinn JL: **Widespread RNA binding by chromatin-associated proteins**. *Genome biology* 2016, **17**:28.
2. Khalil AM, Guttman M, Huarte M, Garber M, Raj A, Rivea Morales D, Thomas K, Presser A, Bernstein BE, van Oudenaarden A *et al*: **Many human large intergenic noncoding RNAs associate with chromatin-modifying complexes and affect gene expression**. *Proceedings of the National Academy of Sciences of the United States of America* 2009, **106**(28):11667-11672.
3. Juan AH, Wang S, Ko KD, Zare H, Tsai PF, Feng X, Vivanco KO, Ascoli AM, Gutierrez-Cruz G, Krebs J *et al*: **Roles of H3K27me2 and H3K27me3 Examined during Fate Specification of Embryonic Stem Cells**. *Cell reports* 2016, **17**(5):1369-1382.
4. Tumes DJ, Onodera A, Suzuki A, Shinoda K, Endo Y, Iwamura C, Hosokawa H, Koseki H, Tokoyoda K, Suzuki Y *et al*: **The polycomb protein Ezh2 regulates differentiation and plasticity of CD4(+) T helper type 1 and type 2 cells**. *Immunity* 2013, **39**(5):819-832.
5. Onodera A, Tumes DJ, Watanabe Y, Hirahara K, Kaneda A, Sugiyama F, Suzuki Y, Nakayama T: **Spatial Interplay between Polycomb and Trithorax Complexes Controls Transcriptional Activity in T Lymphocytes**. *Molecular and cellular biology* 2015, **35**(22):3841-3853.
6. Mozzetta C, Pontis J, Fritsch L, Robin P, Portoso M, Proux C, Margueron R, Ait-Si-Ali S: **The histone H3 lysine 9 methyltransferases G9a and GLP regulate polycomb repressive complex 2-mediated gene silencing**. *Molecular cell* 2014, **53**(2):277-289.
7. Marks H, Kalkan T, Menafra R, Denissov S, Jones K, Hofemeister H, Nichols J, Kranz A, Stewart AF, Smith A *et al*: **The transcriptional and epigenomic foundations of ground state pluripotency**. *Cell* 2012, **149**(3):590-604.
8. Kloet SL, Makowski MM, Baymaz HI, van Voorthuijsen L, Karemaker ID, Santanach A, Jansen P, Di Croce L, Vermeulen M: **The dynamic interactome and genomic targets of Polycomb complexes during stem-cell differentiation**. *Nature structural & molecular biology* 2016, **23**(7):682-690.
9. He A, Ma Q, Cao J, von Gise A, Zhou P, Xie H, Zhang B, Hsing M, Christodoulou DC, Cahan P *et al*: **Polycomb repressive complex 2 regulates normal development of the mouse heart**. *Circulation research* 2012, **110**(3):406-415.
10. Ishak CA, Marshall AE, Passos DT, White CR, Kim SJ, Cecchini MJ, Ferwati S, MacDonald WA, Howlett CJ, Welch ID *et al*: **An RB-EZH2 Complex Mediates Silencing of Repetitive DNA Sequences**. *Molecular cell* 2016, **64**(6):1074-1087.
11. Kaneko S, Son J, Shen SS, Reinberg D, Bonasio R: **PRC2 binds active promoters and contacts nascent RNAs in embryonic stem cells**. *Nature structural & molecular biology* 2013, **20**(11):1258-1264.
12. Rizvi AH, Camara PG, Kandror EK, Roberts TJ, Schieren I, Maniatis T, Rabadan R: **Single-cell topological RNA-seq analysis reveals insights into cellular differentiation and development**. *Nature biotechnology* 2017, **35**(6):551-560.
